# P88 - Protocol implementation: effect on the assistance quality received by patients

**DOI:** 10.1186/2045-7022-4-S1-P143

**Published:** 2014-02-28

**Authors:** Rebeca Mozún-Torrico, Roberto Velasco-Zúñiga, Juan Enrique Trujillo-Wurttele, Sara Martín-Armentia, Leticia González-Martín, Fernando Centeno-Malfaz

**Affiliations:** 1 Hospital Universitario Río Hortega, Valladolid, Spain

## Introduction

The Spanish Pediatrics Emergency Society (SEUP) determined two assistance quality indicators for attendance of asthma exacerbation in Emergency Departments (ED):

1. A validated-score severity evaluation at discharge (standard 100%)

2. Respiratory rate and oxygen saturation registration on the medical record of the patients with chief complaint of breathing difficulty.

In April 2012 a protocol on the management of asthma exacerbation was established in our Emergency Department.

## Objectives

Determine whether the implementation of a protocol meant an improvement in the compliance of the quality standards.

## Methods and material

The study was designed as retrospective and observational. The discharge reports of two cohorts of patients, attended at the Emergency Department of *Hospital Universitario Río Hortega* with chief complaint of asthma exacerbation, were reviewed:

• Group A: patients attended between 1 July 2011 and 31 December 2011.

• Group B: patients attended between 1 July 2012 and 31 December 2012.

## Results

630 medical histories were reviewed in group A and 268 in group B. Different diagnosis such as bronchiolitis and pneumonia were excluded consequently there were 613 patients included in group A and 260 in group B.

**Table 1:** Compliance level for each criterion

	Group A	Group B	p
Registration of oxygen saturation and respiratory rate	176 (28,7%)	154 (59,2%)	<0,001

Registration of severity score	19 (3,1%)	172 (66,2%)	<0,001

Number of parameters achieved • 0 • 1 • 2	435 (70,8%) 163 (26,5%) 16 (2,6%)	59 (22,7%) 76 (29,2%) 125 (48,1%)	<0,001 n.s. <0,001

## Conclusions

A protocol implementation on the management of asthma exacerbation meant an improvement in the quality of attention received. However we are yet far from accomplishing the standards. Quality indicators registration was significantly worse in milder exacerbations. New actions should be designed in order to accomplish the objectives.

